# MtrR Control of a Transcriptional Regulatory Pathway in *Neisseria meningitidis* That Influences Expression of a Gene (*nadA*) Encoding a Vaccine Candidate

**DOI:** 10.1371/journal.pone.0056097

**Published:** 2013-02-08

**Authors:** Jason M. Cloward, William M. Shafer

**Affiliations:** 1 Department of Microbiology and Immunology, Emory University School of Medicine, Atlanta, Georgia, United States of America; 2 Laboratories of Bacterial Pathogenesis, Veterans Affairs Medical Center (Atlanta), Decatur, Georgia, United States of America; Monash University, Australia

## Abstract

The surface-exposed NadA adhesin produced by a subset of capsular serogroup B strains of *Neisseria meningitidis* is currently being considered as a vaccine candidate to prevent invasive disease caused by a hypervirulent lineage of meningococci. Levels of NadA are known to be controlled by both transcriptional regulatory factors and a component of human saliva, 4-hydroxyphenylacetic acid. Herein, we confirmed the capacity of a DNA-binding protein termed FarR to negatively control *nadA* expression. We also found that a known transcriptional regulator of *farR* in *N*. *gonorrhoeae* termed MtrR can have a negative regulatory impact on *farR* and *nadA* expression, especially when over-expressed. MtrR-mediated repression of *nadA* was found to be direct, and its binding to a target DNA sequence containing the *nadA* promoter influenced formation and/or stability of FarR::*nadA* complexes. The complexity of the multi-layered regulation of *nadA* uncovered during this investigation suggests that *N. meningitidis* modulates NadA adhesin protein levels for the purpose of interacting with host cells yet avoiding antibody directed against surface exposed epitopes.

## Introduction

*Neisseria meningitidis* is a Gram-negative obligate human pathogen that colonizes the nasopharynx in 10–35% of adults [1]. For reasons not currently understood, commensal meningococcal (MC) colonization develops into an invasive disease causing septicemia and meningitis in 0.5 per 100,000 persons in the United States and up to 1,000 per 100,000 persons in sub-Saharan African epidemics [2]. The speed of disease progression results in up to 10–15% mortality even with antibiotic therapy [3], while often leaving survivors with permanent neurological complications [4]. Vaccines against the capsular polysaccharide of the most common disease-associated serotypes (A, C, W135, and Y) are available, leaving the hypervirulent and immune-evasive serotype B as a current focus for vaccine research [5].

Adhesion to the mucosal surface of the nasopharynx is the first step in successful colonization, mediated by a variety of factors, with type IV pili [6], [7], [8] and Opa and Opc proteins [9], [10] produced in the greatest abundance. Recently, a non-fimbrial “Oca” family (Oligomeric coiled-coil adhesin) neisserial adhesin termed NadA was identified in 50% of hypervirulent MC capsular serogroup B lineages [11], but not in other capsular serogroup strains. Comprised of a leader peptide, globular “head” domain, α-helix intermediate region, and a C-terminal membrane anchor, NadA forms highly stable multimeric coiled-coil structures along the helical stalk, positioning the globular “head” for host cell interaction [12]. Importantly for consideration as a vaccine candidate, recombinant NadA lacking the C-terminal anchor elicits a bactericidal antibody response with epitopes accessible in encapsulated MC. Although *nadA* allele sequences differ between strains, varied antigen expression, not diversity, influences immune sera titer levels and protection [11]. Accordingly, the identification of factors influencing NadA levels at the gene expression level is critical for optimizing the efficacy of a NadA-targeted vaccine. Furthermore, understanding *nadA* expression may offer clues into the signals involved in converting a passive co-inhabitant of the human mucosal lining into an invasive and fatal septic infection.

**Figure 1:**
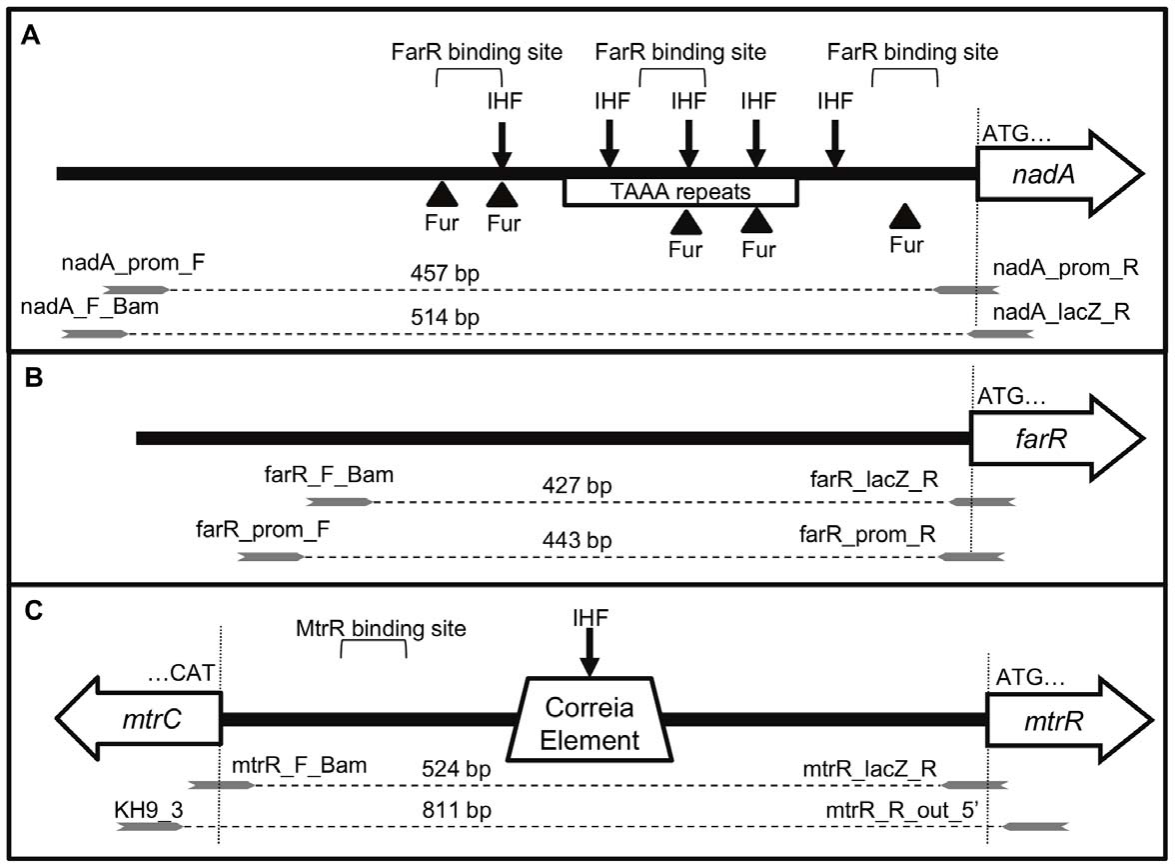
Schematic of *nadA, farR*, and *mtrR* promoter regions used for *lacZ* fusions and DNA probes. Representations of the *nadA* (**A**), *farR* (**B**), and *mtrR* (**C**) promoter regions showing regulatory protein binding sites, intergenic sequences of interest, and primer annealing locations with oligonucleotide sizes. White arrows represent each open reading frame with the translation start codon noted by “ATG” and a vertical line to indicate if a primer overlaps the start codon. Grey arrows represent the respective primers including oligonucleotide sizes with the following nomenclature: DNA probe for EMSAs - “orf”_prom_F & R; promoter region with start codon fused with *lacZ* - “orf”_F_Bam and “orf”_R_lacZ. IHF and Fur are binding sites for integration host factor and ferric uptake regulatory protein, respectively.

MC uses a multi-tiered approach to control *nadA* expression. Maximum levels of the NadA protein are observed in stationary-phase in a growth-dependent manner [11], with expression of *nadA* varying widely among MC strains [13], [14]. Upstream from the promoter are multiple tetranucleotide (TAAA) repeats whose number corresponds with varied *nadA* expression [13], [15]. These repeats are phase variable, likely caused by slipped-strand mispairings during replication [16]. Several regulatory proteins bind to the *nadA* promoter (Figure 1), including integration host factor (IHF) and ferric uptake regulatory protein (Fur), though *nadA* expression is unchanged in a Fur null mutant [14]. Recently, a MarR-family transcriptional regulator, termed FarR and NadR in separate publications [14], [17], was identified as a repressor of *nadA*, further expanding the list of *nadA* regulatory factors. This DNA-binding protein was first identified in the gonococcus (GC) and was shown to repress expression of the *farAB*-encoded efflux pump that is responsible for high levels of fatty acid resistance [18]. In contrast, MC FarR does not affect fatty acid resistance through FarAB, perhaps due to naturally high fatty acid resistance expressed by this pathogen [19]. Interestingly, however, MC FarR does bind to its *farAB* promoter region with relatively high affinity and represses *farAB* expression as shown by RT-PCR [20]. Because FarR regulates expression of *farAB* in both MC and GC, while *nadA* is present only in a subset of MC populations, we will continue to use the nomenclature of FarR for the repressor of *nadA* based on its more universal activity on *farAB* in both GC and MC.

The small molecule 4-hydroxyphenylacetic acid (4HPA) was identified as an inducer or de-repressor of *nadA* by relieving the DNA-binding activity of FarR [14]. Being a colonizer of the oropharynx, MC is washed in saliva, in which 4HPA is a common metabolite [21], possibly leading to increased expression of *nadA* and subsequent invasive disease. Curiously, FarR-controlled targets in GC are directly and indirectly regulated by the TetR family regulator MtrR. Repression of *farR* by MtrR indirectly up-regulates *farAB* [18], while the gene encoding glutamine synthetase (*glnA*) is directly regulated by both FarR and MtrR [22]. Therefore, we questioned whether MtrR similarly affects *nadA* expression in MC, adding to the growing list of regulatory factors targeting *nadA*. Here we confirm that FarR is the primary repressor of *nadA*, yet MtrR, when expressed at elevated levels, directly represses *nadA* as well. Furthermore, DNA-binding and DNase I protection assays suggest that MtrR influences FarR binding at the *nadA* promoter similar to the phenomenon seen in *glnA* expression in GC [22], suggesting a higher complexity to Neisserial regulatory schemes that is conserved across species.

## Results and Discussion

### Control of *nadA, farR*, and *mtrR* expression in MC strain M7

**Figure 2:**
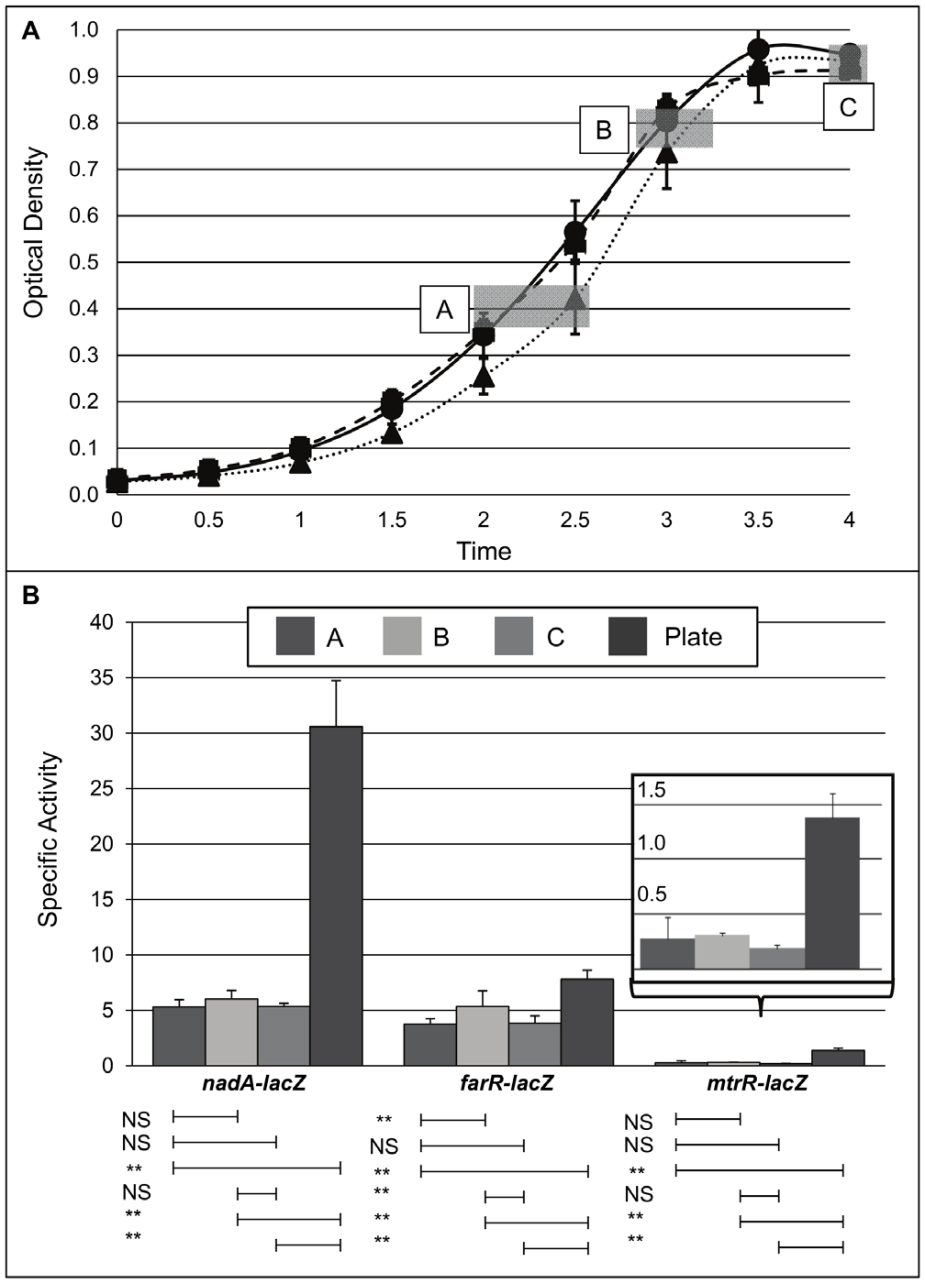
Growth phase-dependent expression of *nadA*, *farR*, and *mtrR* in *N. meningitidis* M7. (A) Growth curve of strain M7 expressing *lacZ* fused to *nadA* (solid line; circle timepoints), *farR* (dotted line; triangle timepoints), and *mtrR* (dashed line; square timepoints) promoter regions measured by OD600 optical density. Boxed A, B, and C; timepoints for sample harvest. (B) Specific activity of β-galatosidase activity of *lacZ* fusions as indicated. Samples harvested from liquid culture at various growth phases (A, B, C) were compared with O/N growth on agar plates. Inset; magnified view of *mtrR-lacZ* expression. NS, not significant; **, P<0.01.

Co-regulation and competitive regulation between FarR and MtrR has been shown previously for multiple targets in GC [18], [22] but not MC. Given previous observations that in MC FarR can regulate *nadA*, we asked if MC MtrR can control *farR* expression. We also tested if MC MtrR could control *nadA* directly. To investigate possible influences of MtrR on *nadA* and/or *farR* expression in MC, the expression profiles for these genes were determined using a promoterless lacZ-fusion expression system (Figure 1) employed previously to monitor gene expression in GC [23]. Using translational *lacZ* fusions to each gene's promoter (ranging from 427 to 524 bp; Figure 1), *mtrR* expression was compared to *farR* and *nadA* across multiple growth phases of broth–grown cultures or from overnight, agar-grown cultures (Figure 1) of MC strain M7, which is a capsule-deficient mutant of strain NMB that is used for biosafety purposes [24]. This was done because earlier work [25] reported that *farR* was maximally expressed between late-log and stationary phase, while *nadA* expression peaks at stationary phase [11]. Importantly, the *lacZ* fusions did not significantly impact growth rates in broth for the M7-derived strains (Figure 2A), suggesting that any expression profile differences are not growth rate-dependent. Aliquots from different growth phases (Figure 2A; boxed A, B, and C) were assessed for β-galactosidase activity and compared against the activity of overnight cultures grown on GCB agar plates (Figure 2B). The results showed that agar-grown MC had higher levels of expression for all three genes compared to broth-grown strains, and this was especially true for *nadA*. With respect to agar-grown cultures, we noted that *nadA* expression was considerably greater than *farR* or *mtrR* with the latter being the most poorly expressed gene (Figure 2B). Based on these results, all subsequent gene expression studies were performed on cultures grown overnight on GCB agar plates.

**Figure 3:**
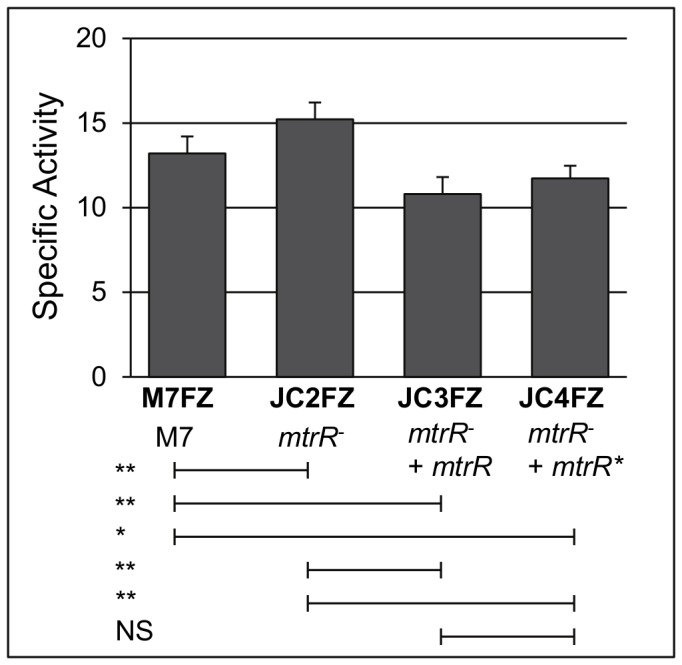
MtrR-regulation of *farR*. Specific activity of β-galactosidase activity of *farR-lacZ* in the strains M7 wild-type, Δ*mtrR*, and Δ*mtrR* complemented with the native and inducible-promoter alleles (superscript asterisk), respectively. NS, not significant; *, P<0.05; **, P<0.01.

**Figure 4:**
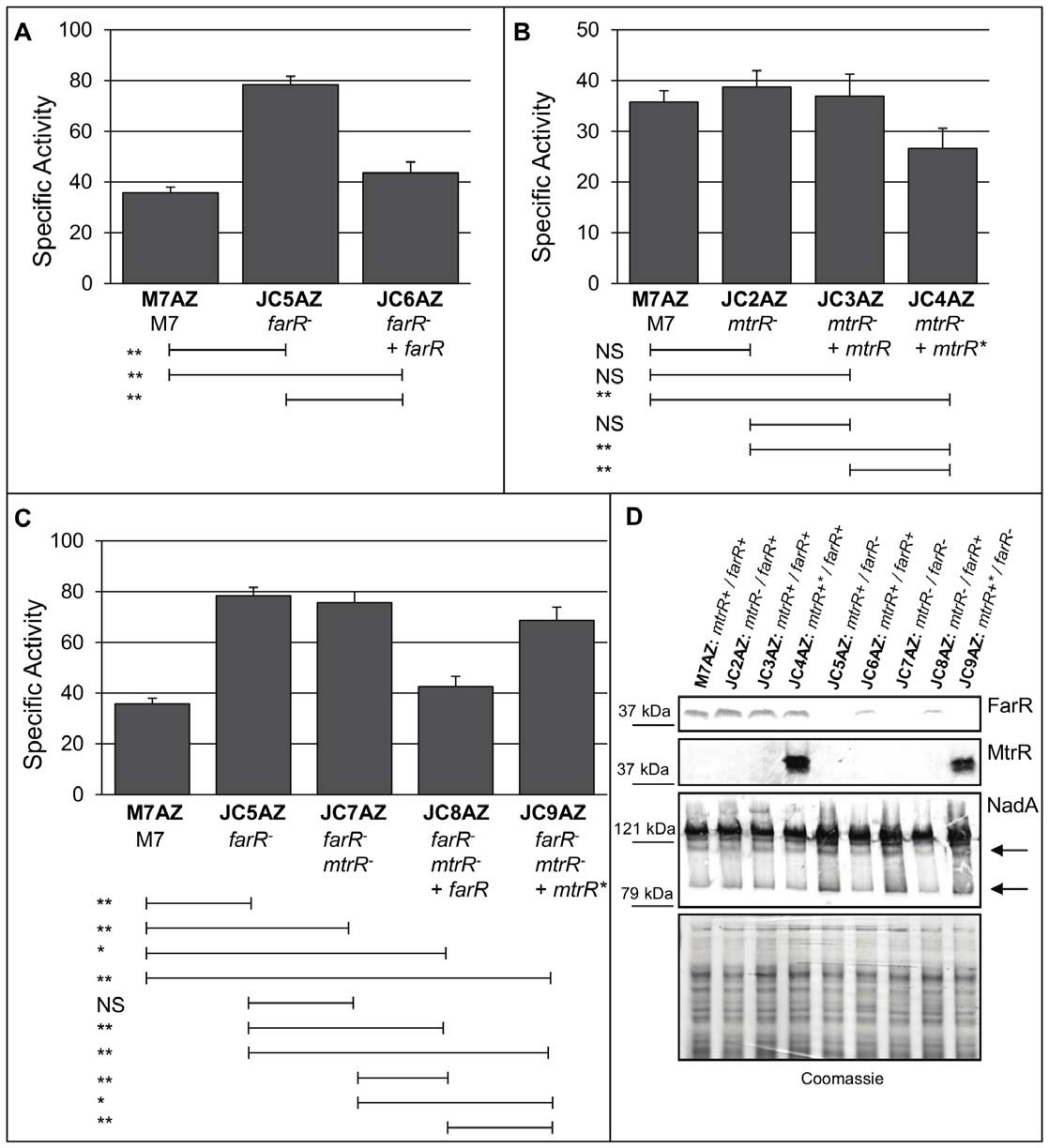
Expression of *nadA* by FarR and MtrR. (A–C) Specific activity of β-galactosidase activity of *nadA-lacZ* in various M7 backgrounds as indicated. Strains over-expressing MtrR marked with a superscript asterisk. NS, not significant; *, P<0.05; **, P<0.01. (D) Western immunoblot analysis of NadA, FarR, and MtrR levels. Protein samples grown overnight on GC agar plates, collected, and analyzed by electropheoresis through 6% (NadA) or 12% (FarR and MtrR) SDS-PAGE gels followed by immunoblot with the respective antisera. Molecular weight standards are listed to the left. Coomassie-stained gel provided for protein level comparison. Arrows represent minor immunoreactive bands used to determine NadA steady-state differences across strains.

Having established that the *lacZ* fusion technology could be employed to monitor gene expression in strain M7, we next asked if MtrR can regulate *farR* as it does in GC and if, in turn, it can modulate *nadA* expression. We first asked if loss of MtrR impacts MC *farR* expression levels and for this purpose constructed an M7 *farR-lacZ* fusion strain (427 bp promoter region; Figure 1) in an *mtrR* null mutant that carries the non-polar *aphA-3* cassette within the *mtrR* coding sequence. With this fusion strain, we noted a small but significant (p<0.01; Figure 3) increase in *farR-lacZ* expression, which was reversed by complementation when the M7 *mtrR* allele was expressed ectopically under its own promoter or an IPTG-inducible promoter. Having observed a significant, albeit modest, influence of MtrR on *farR-lacZ* expression, we next asked if *nadA* expression, known to be negatively controlled by FarR [17], would be impacted due to loss of MtrR using a *nadA-lacZ* fusion (514 bp promoter region; Figure 1). We first confirmed that loss of FarR significantly increased *nadA-lacZ* expression (strain JC5AZ; Figure 4A), and this was reversed by complementation with the wild-type *farR* gene from strain M7 (strain JC6AZ). Interestingly, loss of MtrR alone (strain JC2AZ) resulted in a consistent, albeit small, increase in *nadA-lacZ* expression, though not to significant levels, with restoration of *nadA* to wild-type levels when *mtrR* was expressed ectopically under its own promoter (strain JC3AZ; Figure 4B). Furthermore, complementation with the wild-type *mtrR* sequence under the control of an IPTG-inducible promoter significantly (p<0.01) reduced *nadA-lacZ* expression by 26% (strain JC4AZ; Figure 4B). Based on these results, we concluded that FarR-mediated repression of *nadA-lacZ* was greater than that mediated by MtrR, but that MtrR could impact *nadA* expression by a FarR-independent mechanism. In order to further test this, we compared *nadA-lacZ* expression in a Δ*farR/*Δ*mtrR* double mutant (strain JC7AZ) against the single Δ*farR* mutant (strain JC5AZ). Here, no significant impact on *nadA-lacZ* expression was observed, further supporting a dominant repression of *nadA* by FarR. However, IPTG-induction of *mtrR* in the absence of FarR (strain JC9AZ) did result in a significant reduction in *nadA* (p<0.01) compared to both the Δ*farR/*Δ*mtrR* double mutant (strain JC7AZ) and Δ*farR* mutant (strain JC5AZ; Figure 4C). Taken together, the data suggested that MtrR, when overexpressed, has an influence on *nadA* expression that is independent from FarR-modulated expression in MC strain M7. What effects MtrR may have on *nadA* expression in other MC strains that differ in either *mtrR* or *nadA* expression is not yet clear and the subject of future study.

Western blot analysis was performed to confirm the results observed with translational *nadA* promoter-*lacZ* fusions (Figure 4D). As expected, FarR was absent in the *farR* null mutant strains (JC5AZ, JC7AZ, and JC9AZ), but was present in the complemented mutants that expressed *farR* ectopically (strains JC6AZ and JC8AZ); although FarR levels were reduced in the complemented strains, repression of *nadA* was still evident (Figure 4A, C, D; arrow). NadA migrates in its multimeric form even under denaturing/reducing conditions [11], complicating discrimination of differences in NadA protein levels when analyzing the dominant band alone; therefore, minor NadA-dependent bands, observed elsewhere [20], were used for analysis of steady state levels of NadA (Figure 4D; arrows). Despite repeated attempts, MtrR could not be detected by immunoblot in any strain except when over-expressed (strains JC4AZ and JC9AZ), suggesting that MtrR is maintained at low levels in *N. meningitidis*. This low level of MtrR may explain why enhanced expression of *farR* in the *mtrR* null mutant was modest (Figure 4) and why its ability to repress *nadA* could only be observed when *mtrR* was over-expressed ectopically (strain JC4AZ; Figure 4B). In agreement with the data obtained with the *nadA* promoter translational fusions, steady-state levels of NadA exceeded wild-type in the absence of FarR (Figure 4D; arrows point to minor bands used for comparison). Unfortunately, differences in NadA levels were not discernible when comparing +*mtrR* to Δ*mtrR* strains in the *+farR* (strains M7AZ, JC2AZ, JC3AZ, and JC4AZ) or Δ*farR* (strains JC5AZ, JC7AZ and JC9AZ) backgrounds (Figure 4D). Assessing differences in steady-state levels of NadA in other clinical MC strains following the loss of *mtrR* requires further study.

### FarR and MtrR binding to a target DNA sequence upstream of *nadA*

**Figure 5:**
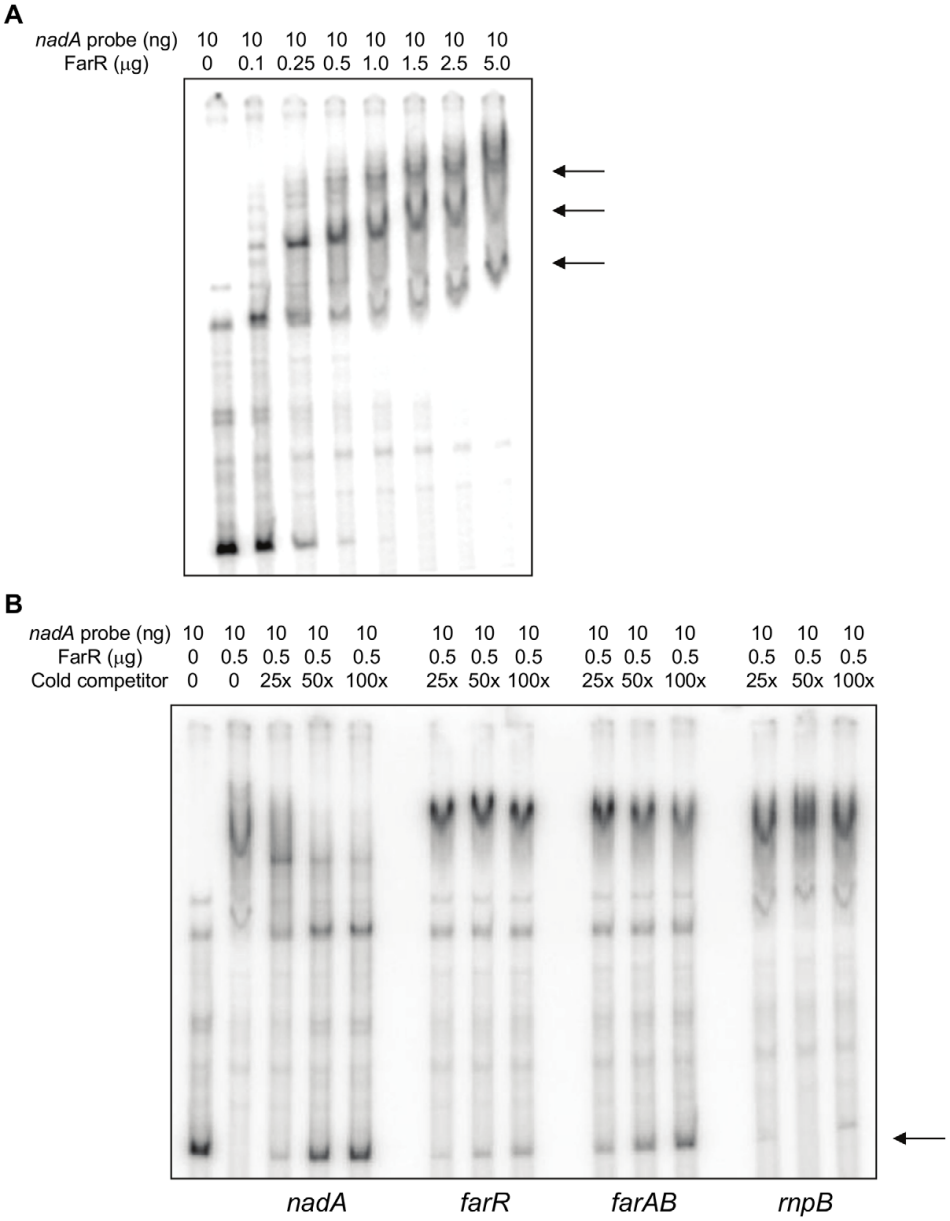
DNA binding properties of FarR-MBP. (A) Successive increases of FarR-MBP incubated with 10 ng *nadA* promoter region to assess binding by gel-shift analysis. Arrows; various complexes of DNA and FarR-MBP. (B) Competition assays. ^32^P-labeled *nadA* promoter (384 bp) was incubated with 0.5 µg FarR-MBP and competed with unlabeled *nadA*, *farR* (333 bp), *farAB* (435 bp), and *rnpB* (354 bp) at 25, 50, and 100 times molar excess of labeled probe (lanes 3 through 14). The competing probe used is listed below each panel. Arrow; ^32^P-labeled probe competed away from FarR-MBP by unlabeled probe. Lane 1, labeled probe alone; lane 2, labeled probe and 0.5 µg FarR-MBP without competitor.

The data obtained using translational fusion strains bearing an over-expressed wild-type *mtrR* allele in an *mtrR* null mutant background indicated that elevated levels of MtrR can exert negative regulatory influences on *nadA* expression in MC strain M7. Based on this hypothesis, we asked if MtrR can bind target DNA sequences upstream of *nadA* by employing electrophoretic mobility shift assays (EMSA) using FarR or MtrR fused to maltose-binding protein (MBP) at their C-terminus;maps describing the various DNA probes used are shown in Figure 1. As described previously [14], FarR was found to bind the target *nadA* sequence (457 bp) in a specific manner with at least three DNA-protein complexes observed (Figure 5A, arrows). Furthermore, the results from binding specificity assays suggested that FarR has greater affinity for the *nadA*-promoter than to its own (443 bp) or *farAB* promoter-bearing sequences (497 bp and shown in Figure 5B); importantly, a probe lacking a FarR-binding site (*rnpB;* 609 bp) was unable to compete with the labeled *nadA* probe for complexing with FarR.

**Figure 6:**
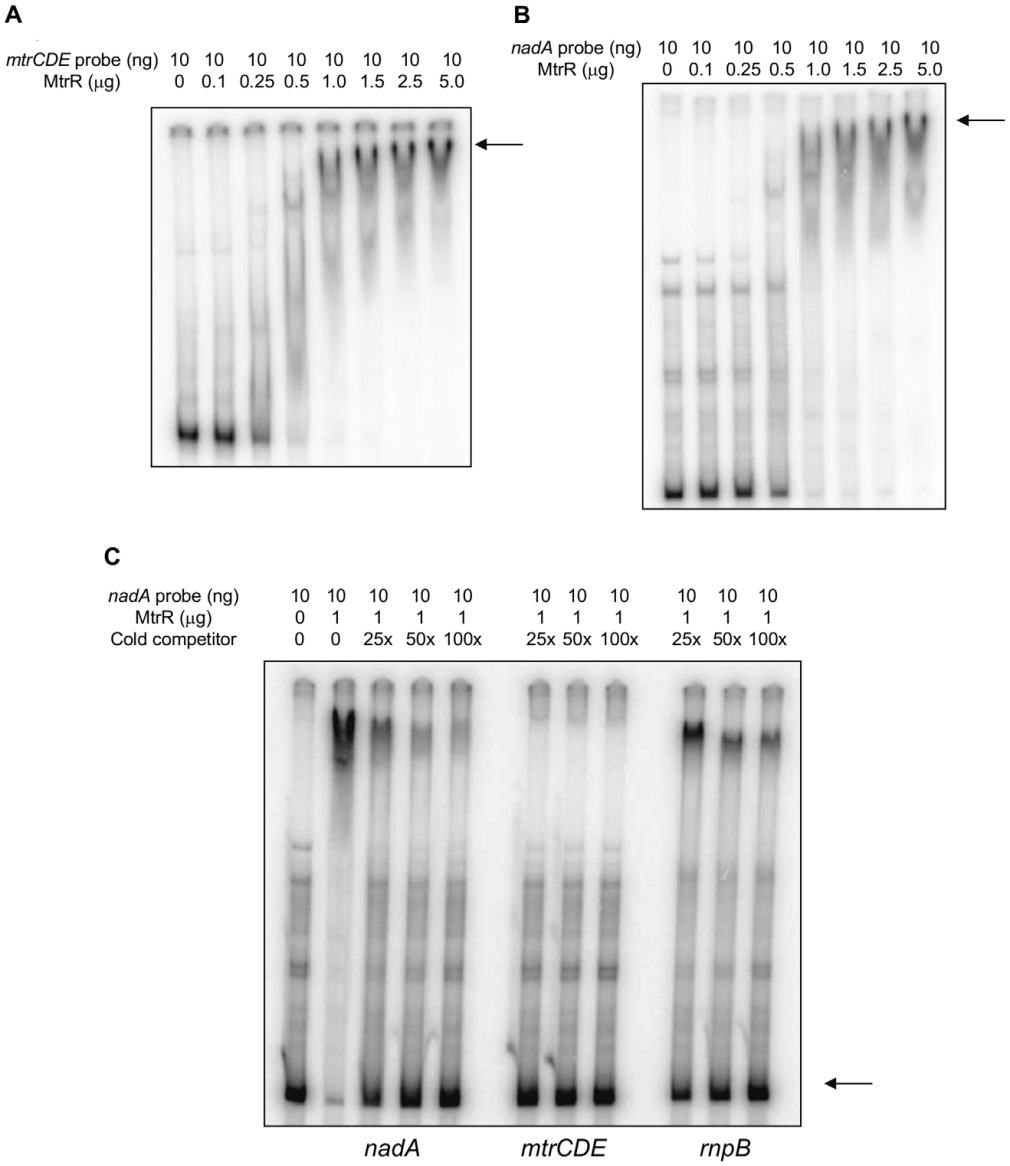
DNA binding properties of MtrR-MBP. Successive increases of MtrR-MBP incubated with 10 ng *mtrCDE* (A) or *nadA* (B) promoter regions to assess binding by gel-shift analysis. Arrow; primary complex of DNA and MtrR-MBP. (C) Competition assays. ^32^P-labeled *nadA* promoter (384 bp) was incubated with 1.0 µg MtrR-MBP and competed with unlabeled *nadA*, *mtrCDE* (552 bp), and *rnpB* (354 bp) at 25, 50, and 100 times molar excess of labeled probe (lanes 3 through 14). The competing probe used is listed below each panel. Arrow; ^32^P-labeled probe competed away from MtrR-MBP by unlabeled probe. Lane 1, labeled probe alone; lane 2, labeled probe and 0.5 µg MtrR-MBP without competitor.

Having verified that the *nadA* probe could be recognized in a specific manner with an MC DNA-binding protein (FarR), we next asked if *nadA* could bind MtrR. First, we confirmed by EMSA the DNA-binding capacity of the MtrR-MBP fusion protein by evaluating its ability to bind a known target DNA sequence, namely the promoter-bearing region upstream of MC *mtrCDE* (811 bp). In GC, MtrR is a repressor of the *mtrCDE*-encoded antimicrobial efflux pump by virtue of its binding between the −10 and −35 promoter elements [26], [27]. We found that as little as 1.0 µg of MC MtrR-MBP incubated with MC *mtrCDE* promoter-bearing probe resulted in virtually a complete shift (Figure 6A; arrow) of the target sequence. Importantly, a similar shifting of the *nadA* probe by MtrR was observed (Figure 6B; arrow). Specificity of MtrR-binding to the *nadA* probe was confirmed by use of competitive EMSA. Although the heterologous unlabeled *rnpB* promoter-bearing probe did to some extent compete for binding, especially at a high concentration (100×), the *mtrCDE* and *nadA* unlabeled probes proved to be more effective competitors at a lower concentration (25×) with the *mtrCDE* probe appearing to be superior (Figure 6C).

**Figure 7:**
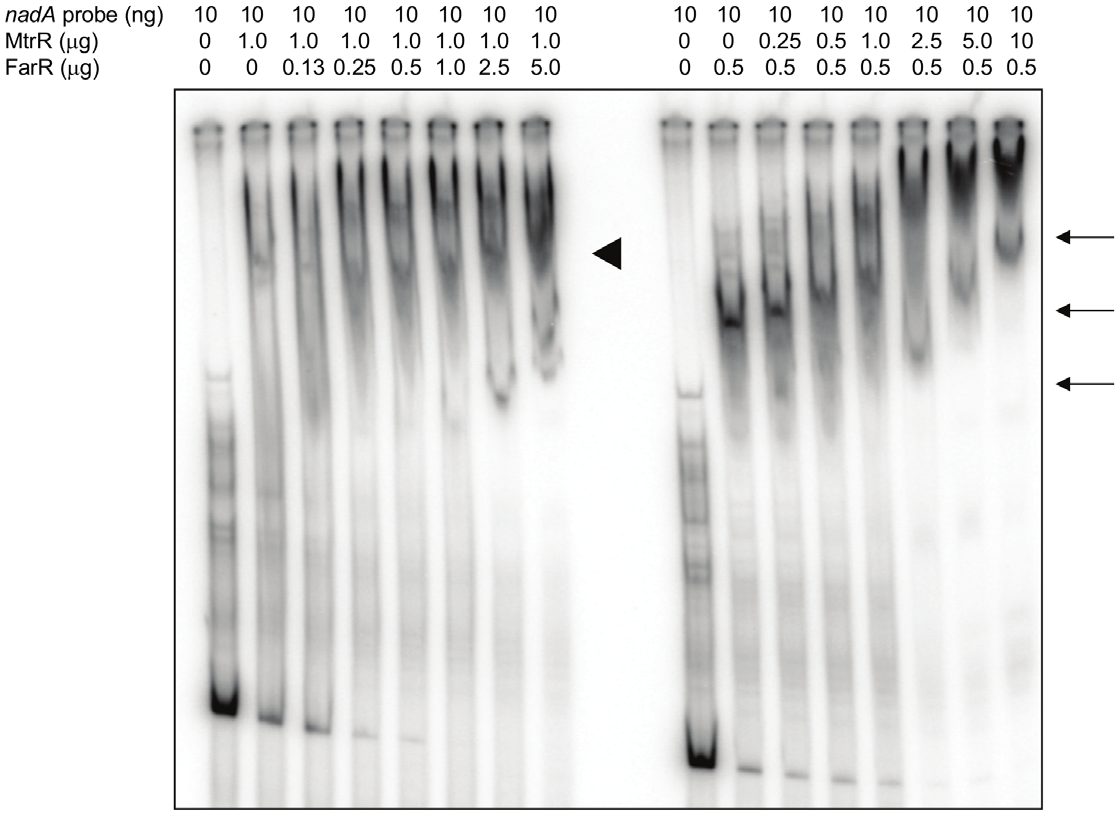
MtrR influences FarR::DNA complexes. Shown is an EMSA evaluating the binding of MtrR–MBP and FarR-MBP to ^32^P-labeled *nadA* promoter based on order of protein introduction. Lane assignments from left to right: probe alone; probe plus 1.0 mg MtrR-MBP and increasing amounts of FarR-MBP (0, 0.13, 0.25, 0.50, 1.0, 2.5, and 5.0 mg); probe alone; probe plus 0.5 mg MtrR-MBP and increasing amounts of FarR-MBP (0, 0.13, 0.25, 0.50, 1.0, 2.5, and 5.0 mg). The arrowhead shows the position of MtrR:DNA complex lacking FarR while the arrows show positions of FarR:DNA complexes lacking MtrR.

The ability of both FarR and MtrR to bind the *nadA* promoter-bearing region in a specific manner was reminiscent of their ability to bind the DNA sequence upstream of *glnA* of GC (22). As the binding of either protein to the *glnA* target can impact binding of the other we asked if a similar situation might exist for the MC-derived *nadA* target. To test this possibility, we pre-incubated the *nadA* probe with a fixed concentration of one protein and then introduced increasing amounts of the second protein. In the absence of a competing protein, both MtrR and FarR exhibited a distinct shift of the probe (Figure 7; arrowhead and arrows, respectively). As FarR-MBP concentrations increased following pre-incubation of *nadA* target DNA with MtrR-MBP, the MtrR shift remained relatively unchanged, suggesting that FarR-MBP does not compete with MtrR-MBP. In contrast, increasing amounts of MtrR-MBP changed the electrophoretic mobility of FarR-MBP:DNA complexes, suggesting that MtrR-MBP can significantly influence the formation or stability of FarR::*nadA* complexes.

**Figure 8:**
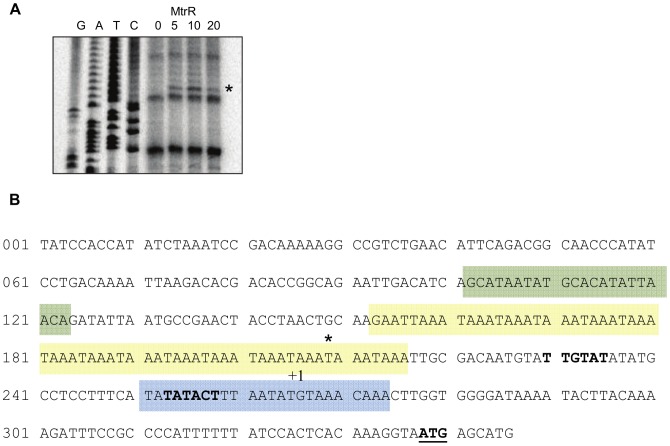
Identification of a DNase I hypersensitive site at the *nadA* promoter in the presence of MtrR. (A) Increasing amounts of MtrR-MBP (0, 1, 5, 10 mg) were incubated with the *nadA* promoter prior to DNase I incubation. Site of DNase I hypersensitivity is denoted with an asterisk. The nucleotide sequence (G, A, T, C) is listed adjacent to the lanes. (B) Nucleotide sequence of the *nadA* promoter. Colored boxes, FarR-MBP binding sites 1, 2 & 3; Asterisk, DNase I hypersensitive site. The transcription start site (+1) and translational start site (ATG, bold and underlined) are indicated.

In order to learn the mechanism by which MtrR could influence the formation of FarR::*nadA* complexes, we used DNase I protection assays to determine if their respective binding sites might be in close proximity. We confirmed FarR-MBP-binding to the three sites (data not shown) described by Metruccio *et al.* [14], which include the −10 promoter and TAAA phase-variable regions (Figure 8B). In repeated DNase I protection assays, clear evidence for a sequence(s) capable of recognizing MtrR could not be obtained. However, in these experiments a DNase I hypersensitive site was identified positioned at the end of the phase-variable TAAA repeats (Figure 8B; asterisk at nucleotide position 209). The presence of this suggests an interaction of MtrR with a sequence upstream of *nadA* that could influence binding of FarR.

While *N. meningitidis* colonizes up to 35% of humans [1], fewer than 1% of the population develops an invasive infection [2], suggesting that the bacterium focuses on a more commensal lifecycle. The ability to effectively transition between passive residence and active infection relies on tight transcriptional regulation involving an array of external and internal control systems. In GC, the transcriptional regulators FarR and MtrR have been well-characterized for their role in antimicrobial resistance, allowing for host persistence [28]. FarR represses expression of the fatty-acid efflux pump FarAB [29]; MtrR represses expression of the *mtrCDE*, which encodes an antimicrobial efflux pump [26], [30]. Recently, MC FarR was shown to repress expression of *nadA* [17], whose gene product is a highly immunogenic adhesin and invasin associated with hypervirulent strains of serotype B MC [11], [12]. Interestingly, GC MtrR has also been shown to repress GC FarR, thereby influencing transcription of *farAB* [18]. As this regulation of a regulator is not unique to GC [22], we explored whether MtrR likewise modulates *farR* in MC, thus affecting *nadA* expression.

Our results suggest that MC employs a dual-repressor approach to control *nadA* expression. Using *lacZ* translational fusions, EMSA, and DNase I protection assays, we confirmed earlier work [14], [17], [25] that FarR is a negative regulator of *nadA* due to its ability to bind target DNA upstream of the coding region (Figure 7B) and affect subsequent expression (Figure 4A). Complicating this regulatory scheme, our results indicate that MtrR can exert an influence on *nadA* directly by interacting with the upstream DNA sequence and indirectly through its ability to reduce *farR* expression (Figure 3). With respect to the first mode of MtrR regulation over *nadA*, our DNA-binding studies indicate that MtrR can bind upstream of this gene in a specific manner (Figure 6C) and can impact the formation and/or stability of FarR::DNA complexes (Figure 7) when its level exceeded that of FarR. The stronger influence of FarR on *nadA* expression is likely due to its ability to recognize three target sites (Figure 7B) while under the conditions employed in the DNase I protection assay. A possible site for MtrR binding could only be surmised by the presence of a DNase I hypersensitive site (Figure 7A). Interestingly, this site is positioned within a tract of tetranucleotide repeats and a FarR-binding site (Figure 7B).

Under what conditions might MtrR-mediated regulation of *nadA* have biologic relevance given the strong regulatory action of FarR? We propose several potential mechanisms: The development of mutations impacting FarR regulation of *nadA* would require alternative mechanisms of transcriptional regulation that could in part be fulfilled by MtrR. Thus, mutations in FarR that reduce its DNA-binding activity or mutations in FarR-binding sites could enhance *nadA* expression unless other regulatory processes are available. Alternatively, mutations that enhance MtrR levels or interactions with *nadA*-binding sites might repress *nadA* expression above that seen by FarR alone. Precedent for clinical isolates of *Neisseria* bearing regulatory mutations impacting gene expression exists in that gonococcal strains isolated from patients frequently contain mutations in *mtrR* and these can cause dysregulation of the *mtrCDE*-encoded efflux pump operon. Furthermore, *cis*-acting regulatory mutations can influence transcription of *mtrR* and/or *mtrCDE* or directly enhance expression of the *mtrCDE-*encoded efflux pump [28], [31], [32], [33]. In M7, *mtrR* expression is typically low, yet overexpression of *mtrR* results in almost 30% repression of *nadA* (Figure 4B and D). Accordingly, it will be important to evaluate MC clinical isolates to determine if they may develop mutations impacting *nadA* expression directly or indirectly; the latter being due to mutations in *farR* or *mtrR*. With the expression of *nadA* varying significantly between MC strains [13], [14], mutations affecting *farR* and/or *mtrR* expression may have more profound effects on *nadA* expression than those observed here. Deletion of the Correia element or IHF binding site upstream from *mtrR* affects expression of *mtrCDE*, which is an MtrR target [32]; Enriquez *et al.* observed several MC isolates with Correia element deletions upstream from *mtrR*, including one serotype B, suggesting that these mutations are not an exception [34]. We propose that this multi-layered regulation of *nadA*, which now includes direct regulation by MtrR, reflects an effort by MC to balance levels of the NadA adhesion important for interacting with host cells yet avoiding potentially protective antibody responses.

## Materials and Methods

### Bacterial strains and growth conditions

All *N. meningitidis* strains listed in Table 1 are derivations of strain M7 constructed for this study; M7 is a stable capsule-negative variant of strain NMB and was used for biosafety purposes. MC were cultured on GCB agar (Difco Laboratories, Detroit, MI) with defined supplements I and II [35] at 37°C under 3.8% (vol/vol) CO_2_. For growth-phase analysis, MC were grown in a shaking incubator at 37°C in GCB broth with sodium bicarbonate and defined supplements I and II as previously described [35].

**Table 1:** Bacterial strains and plasmids used in this study.

Strain	Relevant genotype or remarks	Source
*Neisseria meningitidis*		
M7	Unencapsulated NMB derivative	[24]
JC2	M7 *mtrR::kan^a^*	This study
JC3	As JC2 but *mtrR^+^* delivered using vector pGCC3	This study
JC4	As JC3 but *mtrR^+^* delivered using vector pGCC4	This study
JC5	M7 *farR::spc^b^*	This study
JC6	As JC5 but *farR^+^* delivered using vector pGCC3	This study
JC7	As JC5 but *mtrR::kan*	This study
JC8	As JC7 but *farR^+^* delivered using vector pGCC3	This study
JC9	As JC7 but *mtrR^+^* delivered using vector pGCC4	This study
M7AZ	As M7 but with a *nadA* promoter translationally-fused to *lacZ* and delivered by pLES94 termed *nadA-lacZ*	This study
JC2AZ	As JC2 but with a *nadA-lacZ*	This study
JC3AZ	As JC3 but with a *nadA-lacZ*	This study
JC4AZ	As JC4 but with a *nadA-lacZ*	This study
JC5AZ	As JC5 but with a *nadA-lacZ*	This study
JC6AZ	As JC6 but with a *nadA-lacZ*	This study
JC7AZ	As JC7 but with a *nadA-lacZ*	This study
JC8AZ	As JC8 but with a *nadA-lacZ*	This study
JC9AZ	As JC9 but with a *nadA-lacZ*	This study
M7RZ	As M7 but with an *mtrR* promoter translationally-fused to *lacZ* and delivered by pLES94 termed *mtrR-lacZ*	This study
M7FZ	As M7 but with an *farR* promoter translationally-fused to *lacZ* and delivered by pLES94 termed *farR-lacZ*	This study
JC2FZ	As JC2 but with a *farR-lacZ*	This study
JC3FZ	As JC3 but with a *farR-lacZ*	This study
JC4FZ	As JC4 but with a *farR-lacZ*	This study
*Escherichia coli*		
DH5α	[F^−^ Φ80d*lacZΔ*M15 Δ(*lacZYA-argF*)U169 *endA1 recA1 hsdR17*(r_Κ_^−^ m_Κ_^+^) *deoR thi-1 supE44* λ^−^ *gyrA96 relA1*]	[41]
Top10	[F^−^ *mcrA* Δ(*mrr-hsdRMS-mcrBC*)Φ80 *lacZ*ΔM15 Δ*lacX74 recA1 deoR araD139* Δ(*ara-leu*)*7697 galU galK rpsL* (Str^r^ *endAI nupG*)]	Invitrogen
**Plasmids**		
pCR®2.1-TOPO®	pUC-derived cloning vector; *Amp^R^*	Invitrogen
pUC18K	Source of *aphA-3* cassette; *Kan^R^*	[36]
pHP45Ω	Source of *spc* cassette; *Spc^R^*	[42]
pLES94	pUC18K-derivative allowing a translational fusion to a promoterless *lacZ* fusion and insertion between neisserial *proA* and *proB* genes.	[23]
pGCC3	NCIS vector for insertion of genes directed under their native promoter between neisserial *lctp* and *aspC* genes.	[37]
pGCC4	As pGCC3 except inserted genes are directed under an IPTG-inducible promoter	[37]
pMal-c2	IPTG-inducible expression vector for fusion of proteins to maltose binding protein (MBP) and cytoplasmic expression	NE Biolabs

### Strain construction and verification

For construction of strain JC2, overlapping PCR products were generated to replace *mtrR* with *aphA-3* [36] by allelic exchange at the native *N. meningitidis* locus, conferring kanamycin resistance. Specifically, primers mtrC_R_out_5′ and mtrR_R_Kan_5′ovhg generated product A; kan_F_mtrR_5′ovhg and kan_R_mtrR_3′ovhg generated product B; and mtrR_F_Kan_3′ovhg and NMB1718_FWD generated product C (Table 2). Products A, B, and C were used as template with flanking primers mtrC_R_out_5′ and NMB1718_FWD (Table 2) to generate the final PCR product used for transformation of wild-type M7 with selection on kanamycin at 50 µg ml^−1^. The substitution of *mtrR* with *aphA-3* was verified by PCR (data not shown) and Western blot (Figure 4). For construction of JC5, PCR products generated by FarR_prom_F and FarR _Sma_R (Table 2) were subcloned into vector pCR®2.1 (Invitrogen) generating pJC5α with selection on ampicillin at 100 µg ml^−1^. PCR products from FarR_ Sma_F & FarR_pmalC_Xba_R (Table 2) were purified, digested with *Sma*I, and ligated into pJC5α at the *Sma*I site, generating pJC5β, and sequenced for accuracy. Finally, pHP45Ω was digested with *Sma*I to liberate the *spc* cassette then ligated into the *SmaI* site of pJC5β generating pJC5 used to transform M7 with selection on spectinomycin at 60 µg ml^−1^. The interruption of *farR* with *spc* was verified by PCR (data not shown) and Western blot (Figure 4). Complementation of JC2 and JC5 was accomplished by delivering the parent gene ectopically using vector pGCC3 or pGCC4 [37]. Briefly, primers mtrR_F_Pac_GC3 and mtrR_R_Pme or mtrR_F_Pac_GC4 and mtrR_R_Pme (Table 2) were used to amplify the *mtrR* allele then digested with appropriate restriction enzymes and ligated into pGCC3 and pGCC4, respectively. Similarly, farR_F_Pac_GC3 and farR_R_Pme (Table 2) were used to amplify the *farR* allele for subsequent digestion and ligation into pGCC3. All constructs were verified by sequencing prior to transformation. Transformants were selected on erythromycin at 1 µg ml^−1^.

**Table 2:** Oligonucleotides used.

Primer name	Sequence 5′ → 3′
kan_F_mtrR_5′ovhg	AAA CGC CAT TAT GGC TAA AAT GAG AAT ATC ACC
kan_R_mtrR_3′ovhg	CAA GGC TTG ACT AAA ACA ATT CAT CCA GTA AAA TA
mtrR_R_Kan_5′ovhg	CAT TTT AGC CAT AAT GGC GTT TTC GTT TCG G
mtrR_F_Kan_3′ovhg	ATT GTT TTA GTC AAG CCT TGG TAG CAA TGC
mtrC_R_out_5′	GAA CAG GCG TTT TTG GAT GAT GC
NMB1718_FWD	GCC CAC ATC GTT ATT CTC ATA AAG GC
mtrR_R_Pme	GGG TTT AAA CTT ATT TCC GGC GCA GGT CGG
mtrR_F_Pac_GC3	GCC ATT AAT TAA CCT ATC TGT CTG GTT TGA TGT AAA GGG
mtrR_F_Pac_GC4	GGT TAA TTA ACC GCC CTC ATC AAA CCG ACC
farR_ Sma_F	CTG ATA CAG GCC CGG GAA GCC CTG ATG
farR _Sma_R	CAT CAG GGC TTC CCG GGC CTG TAT CAG
farR_F_Pac_GC3	GGT TAA TTA AGA TGC GGC GGC TTTT GTT TTT TCT GG
farR_R_Pme	GGG TTT AAA CTT ACG AGT TCA ACG CAT CC
nadA_F_Bam	ATA TGG ATC CGT CGA CGT CCT CGA TTA CG
nadA_lacZ_R	ATA TGG ATC CTG TTT CAT GCT CAT TAC C
mtrR_F_Bam	CGG GAT CCC GAG CCA TTA TTT ATC CTA TCT GTC
mtrR_lacZ_R	GGT TGG ATC CAT AAT GGC GTT TTC GTT TCG GG
farR_F_Bam	ATA TGG ATC CGG CGG CTT TTG TTT TTT CTG G
farR_lacZ_R	CGC AGG ATC CGA TTG GGT AGG CAT TGT TCA AG
farR_pmalC_F	ATG CCT ACC CAA TCA AAA CAT GCG
farR_pmalC_Xba_R	TTA CTC TAG ATT ACG AGT TCA ACG CAT CCT CG
mtrR_pmalC_F	ATG AGA AAA ACC AAA ACC GAA GCC
mtrR_pmalC_Xba_R	CAA GTC TAG ATT ATT TCC GGC GCA GGT CG
rnpB1F	CGG GAC GGG CAG ACA GTC GC
rnpB1R	GGA CAG GCG GTA AGC CGG GTT C
farAB_prom_F	ATG TGG GAG GTT TTC GAA CCA CG
farAB_prom_R	CGT GTG CGT ATC CAT AAG ATT GGG
farR_prom_F	CCG CTA TGT AGA GAA TCA AGC GG
farR_prom_R	TTG GGT AGG CAT TGT TTA AGT CTC C
nadA_prom_F	GTC GAC GTC CTC GAT TAC GAA GG
nadA_prom_R	ATG CAT GCT CAT TAC CTT TGT GAG TGG
KH9_3	AGA CGA CAG TGC CAA TGC AAC G
mtrR_R_out_5′	TTG CGG TAA AAG GTT TCC AAG GC

### Construction of *lacZ* reporter fusions, β-galactosidase assay and immunoblot analysis

All *lacZ* fusions used in this study were prepared in pLES94 and performed as described previously [18], [23] using appropriate primers. For *nadA-lacZ*, primers nadA_F_Bam and nadA_lacZ_R were used; for *farR-lacZ*, primers farR_F_Bam and farR_lacZ_R were used (Table 2); and for *mtrR-lacZ*, primers mtrR_F_Bam and mtrR_lacZ_R were used (Table 2) Constructs encoding a *lacZ* fusion were grown overnight on GCB agar with supplements, 1 mM IPTG, and 5 mM 4-hydroxyphenylacetic acid, when appropriate. Cells were harvested directly from plates or used to inoculate GCB broth with appropriate supplements and 1 mM IPTG then grown through stationary phase for growth-phase analysis of protein expression. Cells collected from overnight plates were resuspended in phosphate-buffered saline pH 7.2 (PBS), centrifuged for 2 min at 13,000 rpm, and stored overnight at −20°C after the supernatant was removed. From liquid cultures, 5 mL aliquots were removed at the indicated growth phases (Figure 1) and centrifuged for 15 min at 5,000 rpm. After removing the supernatant, cell pellets were resuspended in PBS, centrifuged for 2 min at 13,000 rpm, and stored overnight at −20°C. To determine β-galactosidase specific activities, cell pellets were resuspended in 50/50 PBS and Z-buffer (60 mM Na_2_HPO4, 40 mM NaH_2_PO4, 10 mM KCl, 1 mM MgCl_2_, 50 mM β-Mercaptoethanol; pH 7.0). After addition of 20 µL 0.1% SDS and 40 µL chloroform, samples were vortexed and incubated at room temperature for 5 min. Protein concentrations were quantified by Nanodrop1000 (Nanodrop Technologies,Wilmington, DE). To 200 µL of Z-buffer, 30 µL of each cell lysate and 70 µL of ONPG (2-Nitrophenyl-β-D-galactopyranoside; Sigma, St. Louis, MO) at a concentration of 4 mg ml^−1^ in Z-buffer was added. Following a color change, the reaction was stopped with 500 µL of 1 M Na_2_CO_3_ solution. The reactions were centrifuged at room temperature for 5 min at 13,000 rpm to remove cell debris and 200 µL of supernatant was transferred to a 96-well microtiter plate and analyzed at 420 nM by a PerkinElmer Victor X3 microplate reader. For data analysis, specific activity was calculated using the formula: {[(OD_420_ * *v*)/(4500 nL nmoles^−1^ cm^−1^×1 cm)]/*t*/mg protein} with *v* being the volume used and *t* being the reaction time. All reactions were performed in triplicate and repeated at least 3 times. Statistical analysis was performed using multivariate ANOVA followed by Tukey HSD post-hoc pairwise comparison using SAS 9.2 software (The SAS Institute, Cary, NC).

Verification of protein absence, overexpression, and comparison between strains were assessed by immunoblot. Total protein was quantified by Nanodrop1000 (Nanodrop Technologies) prior to sodium dodecyl sulfate-polyacrylamide gel electrophoresis (SDS-PAGE) separation [38] and Western immunoblotting [39]. Rabbit polyclonal antibodies were used at the following dilutions: anti-NadA, 1∶2,000; anti-FarR, 1∶5,000; and anti-MtrR, 1∶1,000. Anti-rabbit alkaline phosphatase-conjugated secondary antibody (Bio-Rad Laboratories, Hercules, CA) was used at 1∶7,500.

### Purification of MtrR and FarR

Fusion and purification of *N. meningitidis* M7 MtrR and FarR to maltose-binding protein (MBP) were performed per manufacturer's guidelines (New England Biolabs, Beverly, MA) and as described previously [27] with some exceptions. Primers mtrR_pmalC_F & mtrR_pmalC_Xba_R and primers farR_pmalC_F & farR_pmalC_Xba_R were used to PCR amplify the *mtrR* and *farR* alleles, respectively, before *Xba*I digestion and blunt-end ligation into pMal-c2 (New England Biolabs). Both constructs were sequenced for accuracy. Factor Xa digestion was not performed due to protein stability issues; therefore, all DNA-binding investigations utilizing these proteins maintained an intact MBP fusion, which has been successful in prior investigations [27]. Analysis of eluted fractions by SDS-PAGE revealed a 65-kDa band consistent with the expected size an MtrR-MBP fusion (data not shown). For consistency, *N. meningitidis* M7 FarR was likewise fused to MBP and purified, in which the purified protein band migrated to about 60 kDa (data not shown).

### DNA-binding studies

Electrophoretic mobility shift assays (EMSA) and competitive EMSAs using FarR-MBP and MtrR-MBP were performed essentially as previously described [22], [27], [40] with some modifications. Briefly, unlabeled and ^32^P-labeled probes were generated with the following primers: *nadA*, nadA_prom_F and nadA_prom_R; *mtrCDE*, KH9_3 and mtrR_R_out_5′; *farR*, farR_prom_F and farR_prom_R; *farAB*, farAB_prom_F and farAB_prom_R; and *rnpB*, rnpB1F and rnpB1R (Table 2). Appropriate PCR products were end labeled with [γ-^32^P] dATP using T4 polynucelotide kinase (New England Biolabs, Beverly, MA). The labeled products (10 ng) were each incubated with purified FarR-MBP or MtrR-MBP in a 30 µL reaction volume [10 mM Tris-HCl (pH 7.5), 0.5 mM dithiothreitol, 0.5 mM EDTA, 1 mM MgCl_2_, 50 mM NaCl, 0.05 µg/mL poly(dI-dC)] at room temperature for 30 min. Loading buffer (Epicentre, Madison, WI) was added to each sample then separated by 6% polyacrylamide gel at 4°C, followed by autoradiography. Competitive EMSAs were performed similarly, but unlabeled probes generated from the same primer sets as labeled probes were included.

DNase I protection assays were performed as previously described [22], [27], [40] with slight modifications. Target DNA probes were generated with PCR pimers nadA_prom_F and nadA_prom_R (Table 2). The 5′ end was labeled with T4 polynucleotide kinase as described above for EMSA probes. Purified MtrR-MBP was incubated with target DNA for 15 min at 37°C. Dnase I in loading buffer [10 mM Tris-HCl (pH 7.5), 10 mM MgCl_2_, 4 mM CaCl_2_, 1 mM dithiothreitol, 40% glycerol] was added to each reaction for 1 min at 37°C. The reactions were stopped with DNase I stop buffer (95% ethanol and 7.5 mM ammonium acetate), plunged in an ethanol dry-ice bath for 15 min, and precipitated overnight at −80°C. Pellets were washed in 70% (vol/vol) ethanol, dried, and resuspended in loading buffer (Epicentre). Resuspended reaction mixtures were loaded on 6% denaturing polyacrylamide gel and resolved by autoradiography.
